# Frailty and depressive symptoms in older kidney transplant recipients: opportunities for collaboration between transplant nephrologists and geriatricians

**DOI:** 10.1186/s12877-024-04972-9

**Published:** 2024-05-13

**Authors:** Hana Vankova, Michal Schmalz, Ross Andel, Silvie Rajnochova Bloudickova

**Affiliations:** 1 Department of Internal Medicine, Third Faculty of Medicine, Charles University and University Hospital Královské Vinohrady, Ruska 87, 10000 Praha 10, Prague, Czech Republic; 2 Department of Nephrology, Institute for Clinical and Experimental Medicine, Prague, Czech Republic; 3 Edson College of Nursing and Health Innovation, Arizona State University, Phoenix, AZ USA; 4 Memory Clinic, Department of Neurology, Second Faculty of Medicine, Charles University, Prague, Czech Republic

**Keywords:** Frailty, Kidney transplantation, Posttransplant, Depression, Geriatrics

## Abstract

**Background:**

Frailty is one of the key syndromes in geriatric medicine and an important factor for post-transplant outcomes. We aimed to describe the prevalence of frailty and examine the correlates of frailty and depressive symptoms in older kidney transplant recipients (KTRs).

**Methods:**

This cross-sectional study involved 112 kidney transplant recipients (KTRs) aged 70 and above. Frailty syndrome was assessed using the Fried frailty criteria, and patients were categorized as frail, pre-frail, or non-frail based on five frailty components: muscle weakness, slow walking speed, low physical activity, self-reported exhaustion, and unintentional weight loss. Depressive symptoms were measured using the 15-item Geriatric Depression Scale (GDS). The relationship between frailty and depressive symptoms was evaluated using multinomial logistic regression, with the three frailty categories as the dependent variable and the severity of depressive symptoms as the independent variable, while controlling for age, gender, renal graft function, and time since transplant surgery.

**Results:**

The participants had a mean age of 73.3 ± 3.3 years, and 49% were female. The prevalence of frailty syndrome was 25% (*n* = 28), pre-frailty was 46% (*n* = 52), and 29% (*n* = 32) of the KTRs were non-frail. The mean score for depressive symptoms was 3.1 ± 2.4 points, with 18% scoring above the clinical depression cutoff. Depressive symptoms were positively correlated with frailty (*r* = .46, *p* < .001). Among the frailty components, self-reported exhaustion (*r* = .43, *p* < .001), slow walking speed (*r* = .26, *p* < .01), and low physical activity (*r* = .44, *p* < .001) were significantly positively correlated with depressive symptoms, while muscle strength (*p* = .068) and unintentional weight loss (*p* = .050) were not. A multinomial logistic regression adjusted for covariates indicated that, compared to being non-frail, each additional point on the GDS increased the odds of being pre-frail by 39% (odds ratio [OR] = 1.39, 95% confidence interval [CI] 1.01–1.96) and roughly doubled the odds of being frail (OR = 2.01, 95% CI 1.39–2.89).

**Conclusion:**

There is a strong association between frailty and depression in KTRs aged 70 years and older. Targeted detection has opened up a new avenue for collaboration between geriatricians and transplant nephrologists.

## Background

Frailty, a key syndrome in geriatric medicine, is defined by a reduced physiological ability to withstand stressors. It is associated with a higher risk of negative outcomes, including falls, hospitalizations, higher demands on healthcare resources, and death [1]. Since it was first defined in 2001, the concept of frailty, its assessment, and intervention have been among the main areas of inquiry in the field of geriatrics [2]. In 2018, a consensus conference held by the American Society of Transplantation and the American Society of Nephrology emphasized the significance of frailty in the context of solid organ transplantation in particular [3].

As the number of older patients being referred for kidney transplantation increases, the benefits of transplantation in this population have come under greater scrutiny, making this an increasingly important topic [4]. It is estimated that 20% of kidney transplant recipients (KTRs) are frail [5], and the association between frailty and both short-term and long-term adverse outcomes in kidney transplantation has been well documented [5]. Among the components of frailty, muscle weakness, low physical activity, and slower walking speed have been identified as the most prevalent symptoms in frail KTRs in the United States (US), but data from non-US centers and different patient cohorts are limited [6]. Depressive syndrome has been shown to negatively impact functional status and quality of life not only in geriatric patients but also in KTRs [7]. However, there are few studies that have specifically examined frailty and depression in older kidney transplant candidates and recipients.

In contrast to growing literature on frailty as a predictor in transplant candidates, much less is known about frailty in older KTRs several years after transplantation. Evaluating frailty as a therapeutic target for geriatricians may provide a new perspective for improving the functional status and quality of life of older KTRs. Frailty and depression, both of which are geriatric syndromes, can be modified through effective intervention. This may be achieved through close collaboration between transplant nephrologists and geriatricians in care of transplant patients.

The aim of our study was to determine the prevalence of frailty in older KTRs several years after transplantation, to investigate the factors associated with frailty, and to evaluate the association between depressive symptoms and the degree of frailty.

## Methods

In this cross-sectional study, we evaluated the relationship between frailty and depressive symptoms in 112 KTRs aged 70 years and older, who received kidney transplantation at the Institute for Clinical and Experimental Medicine in Prague, Czech Republic. The inclusion criteria were functioning kidney graft and signed approved informed consent to participate in the study, which was approved by the Ethics Committee of the Institute for Clinical and Experimental Medicine and the Thomayer Hospital. These consecutive cases of patients who agreed to participate in the study underwent a comprehensive examination as part of their regular outpatient check-up between January and June 2021.

Frailty syndrome was identified using the Fried frailty criteria, and patients were classified as frail, pre-frail, or non-frail based on the presence of five frailty components: muscle weakness, slow walking speed, low physical activity, self-reported exhaustion, and unintentional weight loss [2]. Each component was scored as either 1 (present) or 0 (absent). The scoring criteria across the five frailty components are detailed in the Appendix of Fried et al. [2]. Muscle weakness was measured by hand grip strength using a dynamometer with the following sex- and BMI-stratified frailty cutoff criteria: Men: grip strength ≤ 29 Kg for BMI ≤ 24, ≤ 30 Kg for BMI between 24.1 and 28, ≤ 32 Kg for BMI > 28; Women: ≤17 Kg for BMI ≤ 23, ≤ 17.3 Kg for BMI 23.1–26, ≤ 18 Kg for BMI 26.1–29, and ≤ 21 Kg for BMI > 29. Slowed walking speed was measured by walking time of 15 feet with the following sex- and height-stratified frailty cutoff criteria: Men: ≥ 7 seconds for height ≤ 173 cm, ≥ 6 seconds for height > 173 cm; women: ≥ 7 seconds for height ≤ 159 cm, ≥ 6 seconds for height > 159 cm. Low physical activity was measured as kilocalories (Kcal) per week below, with frailty cut-off as < 383 Kcal per work for men and < 270 Kcal per week for women. Unintentional weight loss was measured consistently across all individuals as a self-report of unintentionally losing more than 10 pounds during the past year. Finally, exhaustion was measured as a self-report on two items from the CES-D—“I felt that everything I did was an effort” and “I could not get going”. The items are scored based on “how often in the last week” they felt this way (0 = rarely or none of the time [< 1 day], 1 = some or a little of the time [1–2 days], 2 = a moderate amount of the time [3–4 days], or 3 = most of the time. The exhaustion criterion for frailty was met if a participant answered “2” or “3” on either item. Overall frailty was defined as having ≥ 3 components present, pre-frailty as having 1 or 2 components present, and non-frailty as having 0 components present, according to the original Fried frailty criteria [2]. Depressive symptoms were assessed using the 15-item Geriatric Depression Scale (GDS) [8], a screening tool for measuring depression in older adults composed of fifteen questions (e.g., ‘Do you feel that your life is empty?’) with good reliability (Cronbach’s alpha 0.80 and sensitivity 93%) even in patients with cognitive deficits [8–10]. We used the Czech language version standardized for the Czech Republic [9].

### Statistical analyses

Analyses were performed using SAS software version 9.4 (SAS Institute, Cary, NC). We initially compared KTRs across the three levels of frailty using one-way analysis of variance to compare averages and chi-square tests to compare proportions. We also investigated the intercorrelations among study variables. Pearson correlation coefficients were reported for continuous and dichotomous variables, while Spearman correlation coefficients were used for instances where one or both variables were categorical, such as frailty. Correlations between individual components of frailty and study variables were also examined. The association between frailty and depressive symptoms was evaluated using multinomial logistic regression in the SAS GENMOD procedure, with the three levels of frailty as the dependent variable (non-frail as the reference) and the severity of depressive symptoms as the independent variable. Estimates were adjusted for age, sex, time since transplantation, and kidney function, as measured by the estimated glomerular filtration rate using the CKD-EPI formula. A 2-tailed significance level of 0.05 was used for all statistical tests.

## Results

### Characteristics of the sample

Sample characteristics are presented in Table 1a. The participants had a mean age of 73.3 ± 3.3 years, and 49% were female. Frailty was identified in 25% (*n* = 28) of the participants, while 46% (*n* = 52) were pre-frail, and 29% (*n* = 32) were non-frail.

**Table 1a:** Characteristics of the sample group

	All	Non-frail	Pre-frail	Frail	p-value
Age (years)	73.3 ± 3.3	72.6 ± 3.0	73.2 ± 3.0	74.1 ± 3.9	0.198
Sex (F)	55 (49%)	14 (44%)	22 (42%)	19 (68%)	0.072
Time since transplant (years)	8.6 ± 7.7	5.2 ± 6.4	9.2 ± 7.6	11.4 ± 8.1	0.006
Kidney function (CKD-EPI, ml/s)	47.1 ± 22.4	51.0 ± 20.3	45.5 ± 22.1	45.9 ± 25.4	0.521
Depressive symptoms	3.1 ± 2.4	1.9 ± 1.1	2.8 ± 2.1	5.0 ± 3.0	< 0.001
GDS score of ≥ 5 (N/%)	20(18%)	0	9(17%)	11(39%)	< 0.001
Unintentional weight loss (N/%)	15 (13%)	0	4(8%)	11(39%)	< 0.001
Muscle weakness (N/%)	30 (26%)	0	16(30%)	14(50%)	< 0.001
Slow walking speed (N/%)	25 (22%)	0	5 (9%)	20(71%)	< 0.001
Self-reported exhaustion (N/%)	52 (46%)	0	28(53%)	24(86%)	< 0.001
Low physical activity (N/%)	38 (34%)	0	13(25%)	25(89%)	< 0.001

*Note* P-values were calculated using a one-way analysis of variance to compare mean differences and a chi-square test to compare proportions

The mean GDS score was 3.3 ± 2.4. A GDS score of ≥ 5, indicating a potential clinical burden of depressive symptoms, was found in 18% of the participants. The non-frail subgroup had a mean GDS score of 1.9 ± 1.1, while the frail subgroup had a mean score of 5.0 ± 3.0. In terms of individual frailty components in our sample group, slow walking speed was present in 22%, low physical activity in 34%, low muscle strength in 26%, unintentional weight loss in 13%, and self-reported exhaustion in 46%. Mean values for handgrip strength characterizing eventual muscle weakness were 223 ± 4.4 in women and 35.1 ± 5.9 in men. The frequency of individual frailty components categorized by sex is presented in Table 1b.

**Table 1b:** Individual components of frailty in the context of gender

	All	F	M	p-value
Non-frail (N/%)	32 (29)	14 (25)	18 (32)	
Pre-frail (N/%)	52 (46)	22 (40)	30 (53)	
Frail (N/%)	28 (25)	19 (35)	9 (16)	0.72
Unintentional weight loss (N/%)	15 (13)	11 (20)	4 (7)	0.04
Muscle weakness (N/%)	30 (26)	16 (27)	14 (25)	0.74
Slow walking speed (N/%)	25 (22)	15 (27)	10 (18)	0.22
Self-reported exhaustion (N/%)	52 (46)	29 (53)	23 (39)	0.13
Low physical activity (N/%)	38 (34)	22 (40)	16 (28)	0.18

*Note* P-values were based on a chi-square test for comparison of proportions

### Association between frailty and depressive symptoms

Depressive symptoms were found to be positively associated with frailty (*r* = .46, *p* < .001). Among the frailty components, depressive symptoms were positively associated with slow walking speed (*r* = .26, *p* < .01), low physical activity (*r* = .44, *p* < .001), and self-reported exhaustion (*r* = .43, *p* < .001), while the associations with muscle strength and unintentional weight loss were not significant. Intercorrelations among study variables are presented in Table 2.

**Table 2:** Intercorrelations among study variables

	Mean ± SD or n(%)	1.	2.	3.	4.	5.	6.	7.	8.	9.	10.	11.
1. Age	73.3 ± 3.3	1.00										
2. Sex	50 (49%)	0.02	1.00									
3. Time since transplant	8.6 7.7	0.30***	0.02	1.00								
4. Kidney function	47.1 22.4	0.09	0.18	− 0.14	1.00							
5. Depressive symptoms	3.1 ± 2.4	0.02	− 0.16	0.05	− 0.05	1.00						
6. Categories of frailty	28 (25%)	0.17	− 0.17	0.30***	− 0.09	0.46***	1.00					
7. Muscle weakness	29 (26%)	0.11	− 0.03	0.25**	− 0.05	0.19	0.42***	1.00				
8. Slow walking speed	25 (22%)	0.28**	− 0.12	0.10	0.08	0.26**	0.61***	0.12	1.00			
9. Low physical activity	38 (34%)	0.11	− 0.13	0.19	− 0.08	0.44***	0.68***	0.14	0.43***	1.00		
10. Self-reported exhaustion	51 (46%)	0.03	− 0.14	0.17	− 0.14	0.43***	0.63***	0.03	0.33***	0.29**	1.00	
11. Unintentional weight loss	15 (13%)	0.07	− 0.19	0.20	− 0.04	0.17	0.41***	0.13	0.23*	0.22*	0.11	1.00

*Note* SD = standard deviation; depressive symptoms are based on the 15-item Geriatric Depression Scale score. *p < .05, **p < .01, ***p < .001

According to a fully adjusted multinomial logistic regression model (Table 3), each additional point on the GDS increased the odds of being pre-frail by 39% (odds ratio [OR] = 1.39, 95% confidence interval [CI] 1.01–1.96) and nearly doubled the odds of being frail (OR = 2.01, 95% CI 1.39–2.89), compared to being non-frail. The effect of depression on frailty was found to be independent of both kidney graft function and time since transplantation.

**Table 3:** Multinomial logistic regression assessing the risk of pre-frailty or frailty as a function of study variables

95% confidence interval				
	Odds ratio	Low	High	p-value
Age				
Pre-frailty	1.032	0.877	1.215	0.703
Frailty	1.107	0.908	1.349	0.315
Sex				
Pre-frailty	1.059	0.399	2.811	0.908
Frailty	0.394	0.109	1.417	0.154
Time since transplant (years)				
Pre-frailty	1.079	0.998	1.167	0.056
Frailty	1.126	1.026	1.235	0.012
Kidney function				
Pre-frailty	0.992	0.970	1.015	0.502
Frailty	0.996	0.967	1.026	0.812
Depressive symptoms				
Pre-frailty	1.394	1.006	1.933	0.046
Frailty	2.008	1.393	2.894	< 0.001

*Note* Being non-frail was reference

### Additional clinical characteristics

Additional variables were available that did not correlate with our outcome and were therefore not meaningful methodologically, but they help us provide additional, clinically meaningful information about our sample. The etiology of end stage renal disease for the whole sample was diabetes mellitus 15%, hypertension 8%, polycystic kidney disease 20%, glomerulonephritis 35%, other 22%. Average dialysis vintage prior transplant was 2.3 ± 1.6 years. Living donor transplantation was performed in 7 (6%) cases, 3 in non-frail and 4 in pre-frail subgroup. In majority of patients, it was first kidney transplantation, while in six patients it was repeated transplantations, in five cases second transplantation (4 in pre-frail subgroup, 1 in frail subgroup) and in one case third transplantation (frail subgroup). Concerning the comorbidities, diabetes mellitus was present in 40% non-frail, 43% pre-frail and 43% frail participants, cardiovascular disease was present in 28% non-frail, 32% pre-frail and 25% frail participants in the time of our analysis. Concomitant immunosuppressive therapy consisted mainly of a combination of calcineurin inhibitor (CNI) and antimetabolite. All subgroups were managed with CNI in 84.1% on average, whereas antimetabolite was administered in 64.2% in pre-frail and in 71.4% in frail compared to 81.3% in non-frail participants.

Polypharmacotherapy was observed: median number of used medications was very high for the whole sample (14) as well as for all subgroups: 13 for non-frail (mean 12.1 ± 2.9), 14 for pre-frail (mean 13.3 ± 2.8) and 14 for frail participants (mean 14.7 ± 3.1).

Although the differences in medication use across the three frailty groups were relatively insubstantial, in an analysis of variance, the non-frail group appeared to take significantly fewer medications than the frail group (p,0.05). Therefore, we considered the medication count as a covariate in the main analyses and the pattern of our results remained unchanged.

## Discussion

Frailty is a complex syndrome that is characterized by a reduced ability to withstand stressors due to decreased physiological reserves, particularly in older patients. Although the diagnosis and management of frailty have typically been considered a subdomain of geriatrics, other medical specialists have also begun to address this issue due to the increasing age of patients.

In the field of kidney transplantation specifically, frailty has become a frequently discussed issue due to the increasing number of older patients being referred for transplantation. The prevalence of frailty among individuals with chronic kidney disease (CKD) increases with the stage of CKD and can reach up to 35% among patients undergoing hemodialysis [10]. Frailty in kidney transplant patients is associated with an increased risk of falls, hospitalizations, poor cognitive function, reduced quality of life, and higher mortality [10]. Studies, primarily from the US and Asia, have found a 20% prevalence of frailty among kidney transplant recipients and have confirmed its association with negative post-transplant outcomes such as delayed graft function, prolonged hospital stay, immunosuppression intolerance, decreased quality of life, and mortality [11]. As a result, identifying frailty in kidney transplant candidates has become an important factor in both the transplant evaluation process and prehabilitation for patients accepted for transplantation to improve post-transplant outcomes.

It is commonly believed that good renal graft function can prevent the occurrence of frailty and depression in KTRs, regardless of the time since transplantation. However, most studies have focused on the prevalence of frailty in kidney transplant candidates or in KTRs at the time of transplant surgery and during the early post-transplant period. A recent meta-analysis of eighteen studies, fourteen of which were conducted at Johns Hopkins Hospital, estimated that the overall prevalence of frailty in kidney transplant candidates aged 44 to 54 years was 17.1% [11].

In a US-based study with a cohort aged 53.3 ± 14.2 years, it was found that 19.8% of patients were frail at the time of kidney transplant surgery, and 17.2% remained frail three months after transplantation [12]. Similarly, in a Brazilian study with a cohort aged 44.9 ± 12.2 years, out of 15.6% of patients who were frail at the time of kidney transplant surgery, 4.7% remained frail 12 months after transplantation, with weight loss being the most frequently reduced PFP component [6].

While there is some understanding of the prevalence of frailty in CKD patients and KTRs in the early post-transplant period, we found only two studies focusing on long-term post-transplant observation. One study by the McAdams-DeMarco group found that in KTRs with an average age of 52.7 years, the likelihood of frailty decreased (aOR 0.96) during the first 2.5 years after transplantation but increased thereafter [13]. Another single-center study from the Netherlands using the Groningen Frailty Indicator (GFI) observed a decline in frailty occurrence in 20% of KTRs 22.8 ± 8.3 months after kidney transplantation [14].

In our study, we examined the relationship between frailty and depressive symptoms in older KTRs, who are the most vulnerable patient population. All participants were over seventy years of age (73.3 ± 3.3 years) and had undergone kidney transplant surgery an average of 8.6 ± 7.7 years prior. We found that the prevalence of frailty and pre-frailty was 25% and 49%, respectively.

In our study, we used the Physical Frailty Phenotype (PFP) to assess frailty, as it is the most commonly used tool in KTR population studies [11]. While there is complete agreement in using scores of three to five positive components to define the frailty category, there is some disagreement in the cut-off points for the robust/non-frail or pre-frail/intermediately frail categories in some KTR papers, which differ from the standard PFP calculation. Some studies combine scores of 0 and 1 into a single robust/non-frail category and only consider a score of 2 as pre-frail, arguing that few patients with ESRD score zero and fulfill none of the frailty components [12, 15, 16].

However, a Spanish study that specifically addressed this methodological question found that the presence of even a single PFP frailty component (score 1) has an independent impact on KTR survival, a correlate of frailty, after transplantation [17]. Based on this finding, we used the original PFP criteria as in geriatrics, where a patient is considered robust/non-frail only if no PFP components are present [2].

The quality of frailty evaluation methodology is crucial for accurate interpretation [18]. In a multicenter US study, the use of a validated frailty assessment tool was associated with improved waitlist survival in older kidney transplant candidates, and centers that used frailty assessment at admission for transplantation were more likely to have better graft survival rates [19]. Several components of comprehensive geriatric assessment, such as ADL and IADL disability, have been identified as correlates of frailty in KTRs [15].

Our findings highlight the strong relationship between frailty and depressive symptoms in older KTRs. Notably, each additional point on the GDS scale approximately doubled the odds of being frail. The occurrence of depressive symptoms was independent of renal graft function and time since transplantation. Among the frailty components, we found a positive correlation between depressive symptoms and not only self-reported exhaustion but also slow walking speed and low physical activity.

Existing evidence suggests that post-transplant depression, which affects up to 20% of KTRs, is also associated with non-adherence to medication, graft failure, and a return to dialysis therapy [7]. Even minor depression can significantly impair functional status and quality of life, regardless of preserved renal graft function [20]. Factors such as malnutrition, low physical activity, and polypharmacy have been identified as influencing the occurrence of frailty in the long term [21]. Targeted identification and intervention of these factors may positively impact long-term renal transplantation outcomes.

Best practice guidelines recommend a holistic approach to frailty intervention based on comprehensive geriatric assessment [22]. The results of such an assessment can be beneficial, but their interpretation in context is crucial [23]. The greatest benefit of frailty evaluation would be fully achieved if it were followed by a clinical process in which a geriatrician participates in interpreting the multidimensional assessment results, identifying underlying causes, and estimating available resources to organize a personalized, multidisciplinary intervention plan [24].

In light of the mutual interaction between frailty components, depressive syndrome, and renal transplantation outcomes, it can be strongly recommended that transplant nephrologists and geriatricians collaborate.

The results of the study should be interpreted with caution due to a few limitations, including its cross-sectional design and limited sample size. The findings are limited to use of GDS scale, whereas diagnoses of psychiatric disorders, anxiety, and apathy, which would have been useful in the context of this study, were not available as well as the state of the cognition. These factors would enrich future research in this area. In addition, although the time since transplantation is not commonly included as a covariate in this line of research, we felt compelled to control for this variable given the variability in time since transplantation in our sample and the significant correlation with the outcome. Although removing it did not alter the association between depressive symptoms and frailty appreciably (OR = 1.45, 95% CI 1.04–2.02 for pre-frailty and OR = 2.06, 95% CI 1.43–2.98 for frailty). Finally, as Table 3 shows, we found that each additional year from transplantation was associated with 8% greater odds of pre-frailty (OR = 1.08, 95% CI 1.00-1.17, *p* = .012) and 13% greater odds of frailty (OR = 1.13, 95% CI 1.03–1.24) compared to non-frailty. The association between time since transplantation and frailty outcomes may deserve attention in future research. Finally, not all variables that were available were ultimately presented in the results. These additional variables helped describe our sample but were not correlated with our outcome.

A notable strength of this study is its focus on post-transplant frailty and depression in the most vulnerable older KTRs over the long-term, using standardized assessment tools. This is the first study to address the suggested need for closer monitoring of KTRs for worsening frailty after 2.5 years posttransplant, despite improvements short-term after transplant surgery [13]. Further research is needed to determine the effect of intervention programs on depression and frailty domains in older KTRs.

## Conclusion

The prevalence of frailty is high among KTRs, and there appears to be a significant correlation between depressive symptoms and frailty in the KTR population aged 70 and above. Targeted interventions addressing both frailty and depressive symptoms are key competencies in geriatric medicine, providing an opportunity for beneficial collaboration between geriatricians and transplant nephrologists.

## Data Availability

Data available from the corresponding author on reasonable request.

## Abbreviations

KTRs: Kidney transplant recipients
GDS: Geriatric Depression Scale
PFP: Physical Frailty Phenotype
